# Severe pre-eclamptic women with headache: is posterior reversible encephalopathy syndrome an associated concurrent finding?

**DOI:** 10.1186/s12884-020-03017-4

**Published:** 2020-06-01

**Authors:** An-Shine Chao, Yao-Liang Chen, Yao-Lung Chang, Angel Chao, Seng-Yuan Su, Tzu-Hao Wang

**Affiliations:** 1 Department of Obstetrics & Gynecology, Chang Gung Memorial Hospital and Chang Gung University, 5, Fu-Shin street, Kei-Shan, Tao-Yuan, 333 The People’s Republic of China; 2 Department of Obstetrics & Gynecology, New Taipei Municipal Tu Cheng Hospital, 6, Sec.2, Jincheng Road, Tu Cheng, New Taipei City, 236 Taiwan; 3 Department of Diagnostic Radiology, Keelung, Chang Gung Memorial Hospital and Chang Gung University, 222, Maijin Road, Keelung, Taiwan; 4 Department of Obstetrics & Gynecology, China Medical University HsinChu Hospital, Taiwan, 199, Sec., 1, Xinglong road, HsinChu, 302 Taiwan

**Keywords:** MRI (magnetic resonance imaging), Neuroimaging, Severe pre-eclampsia, Posterior reversible encephalopathy syndrome (PRES)

## Abstract

**Background:**

A high incidence of posterior reversible encephalopathy syndrome (PRES) has been observed in women with eclampsia on imaging. However this association was documented mostly after convulsions occurred. This study aimed to detect the development of PRES using magnetic resonance imaging (MRI) in women with severe preeclampsia and headache, and evaluate the clinical and radiological findings in obstetric outcomes.

**Methods:**

A prospective single-center cohort study comprising 20 pregnant women with severe pre-eclampsia related headache was conducted using Numeric Rating Scale (NRS) score of ≧4. Additionally, non-contrast brain MRI was used to detect PRES and related radiological central nervous system (CNS) abnormalities.

**Results:**

Patients were enrolled at a mean gestational age of 32 weeks (range 29–38 weeks). Two women were unable to complete the scanning. Of the 18 MRI scans, 15 (83%) revealed abnormal findings. One patient developed an altered mental state and diffuse PRES, with the occipital, temporal, thalamus, and basal ganglia, the brain stem, and the cerebellum being affected. Two patients had abnormal susceptibility-weighted imaging (SWI) findings, indicating micro-hemorrhages. The majority (12 cases, 66%) of the patients had abnormal cortical hyperintensities in the occipital and temporal lobes. Only three patients had normal MRI pictures. None of the women had eclampsia occurred during the peripartum period, and only one unrelated neonatal death due to congenital anomalies.

**Conclusion:**

A high incidence of abnormal cortical hyperintensity changes at locations typical for PRES on MRI was noted in women with severe pre-eclampsia and headache. These early hypertensive neurological signs allowed prompt and efficient obstetrical management, to prevent the development of eclampsia and PRES.

## Background

Posterior reversible encephalopathy syndrome (PRES) is an increasingly recognized condition associated with eclampsia observed through radiological findings. It includes convulsion and a combination of conscious and visual disturbance, leading to abnormal neuroimaging findings [1–5]. PRES is also known as acute hypertensive encephalopathy or reversible posterior leukoencephalopathy. Thus, acute hypertension plays a major role in the imaging presentation of cortical changes on magnetic resonance imaging (MRI), which is completely different from that observed in patients with chronic hypertensive encephalopathy. However, limited neuroimaging information regarding severe pre-eclampsia is available [5, 6]. Headache-associated severe preecalmpsia is an obstetrical emergency. More information on preventing deterioration into eclampsia can reduce both maternal and fetal mortality. However, no consistent protocol on whether a patient must undergo neuroimaging is available, which introduces a selection bias with regard to the patients who are neuroimaged [6, 7]. Currently, a conservative approach is frequently adopted for severe pre-eclampsia because of the fear of the adverse effects of treatment and etiological investigations on the mother and unborn fetus [6]. The present study aimed to detect early CNS changes, by magnetic resonance imaging, in women with severe preeclampsia having headache. An attempt shall be made to elaborate the known pathophysiological aspects for considering the observed changes.

## Methods

### Study population

Patients presenting with severe features of pre-eclampsia and headache between July 2013 and June 2014 in a single institute, a tertiary hospital, were enrolled in this study. Approval for this study was obtained from the ethics committee of Chang Gung Memorial Hospital (reference number 100-4462A3), and written consent from all participants. Severe pre-eclampsia was defined as having systolic blood pressure of > 160 mmHg, diastolic blood pressures of > 110 mmHg, and usually having proteinuria of 300 mg in 24 h after the 20th week of gestation, which may be accompanied by thrombocytopenia, impaired liver function, nausea and vomiting, renal insufficiency, pulmonary edema, and new-onset cerebral or visual disturbance [5]. The headache intensity was assessed by NRS, wherein a score of ≥4 indicated a requirement of pain relief treatment. The exclusion criteria were as follows: (a) receiving antiepileptic therapy; (b) history of CNS surgery; (c) seizure or previously known psychiatric or neurologic disorders; (d) gestational age of less than 20 weeks; (e) a known history of underlying clinical conditions linked to PRES, including infection, renal disease, auto-immune disorders, and taking immunosuppressive medications. All patients were follow- up until December 2016.

Standard obstetric care was provided at admission with carefully monitoring of fetal heart rate and adequate controlling of blood pressure (BP), which mandatory. Patients were intravenously (IV) administrated 4 g of MgSO4 upon their arrival and were provided a maintenance dosage through the postpartum period to prevent seizure attack, and antihypertensive prescriptions of labetalol (10 mg IV in progressively increasing doses [20, 40, and 80 mg] every 10–15 min to a maximum dose of 200 mg) or hydralazine (10 mg IV every 20 min to a maximum dose of 30 mg) was prescribed to reach the goal of BP of ≦160/100 mmHg. Additionally, electronic fetal heart rate tracing was continuously performed to monitor adequate uteroplacental flow for the fetus [5]. MRI was performed after initial stabilization or was avoided due to critical status. Prompt delivery was performed after maternal stabilization for patients with a gestational age more than 34 weeks and after antenatal corticosteroid treatment if the gestational age was less than 34 weeks. Careful in-hospital expectant management was provided to patients with a gestational age less than 28 weeks.

### Clinical data collection

Baseline characteristics including maternal age, parity, gestational age of presentation, history of pre-eclampsia or eclampsia, route of delivery, and fetal outcome were analyzed. Patients were follow-up through outpatient medical visits or by telephone.

### MRI imaging protocol

The MRI scans were conducted at Chang Gung Memorial Hospital using a 3.0-T MRI GE™ scanner (Discovery MR 750, GE Healthcare, Waukesha, WI, USA). Brain MRI was performed within 24–48 h after the admission, or as early as possible, within 4 h, if the patient experienced an episode of malignant hypertension or had a new onset of severe headache or blurred vision. Axial fluid-attenuated inversion recovery (FLAIR) and susceptibility-weighted imaging (SWI) were the first two sequences for screening the brain status within the first 5 min for evaluating the PRES severity or evidence of microbleeds, which may require appropriate time to secure the reversibility of neurological functions. No administration of Gadolinium is provided in this IRB. The participants had their heads restrained by a plastic fixation pad. All the women were provided with ear plugs. Each scan was performed within 35–40 min.

MRI scans were performed and reviewed by two neuroradiologists to form a consensus so as to minimize interinterpretational differences. The two radiologists reviewed all the 18 cases. The diagnosis of PRES and abnormal findings were made based on the standard radiological criteria [8, 9], which were noted as subcortical and gyral FLAIR hyperintensities that became more diffused with an increase in the extent of edema.

### Statistical analyses

Statistical values of demographic characteristics are expressed as means (range), and categorical variables are expressed as numbers and percentages.

## Results

### Patient characteristics

**Fig. 1:**
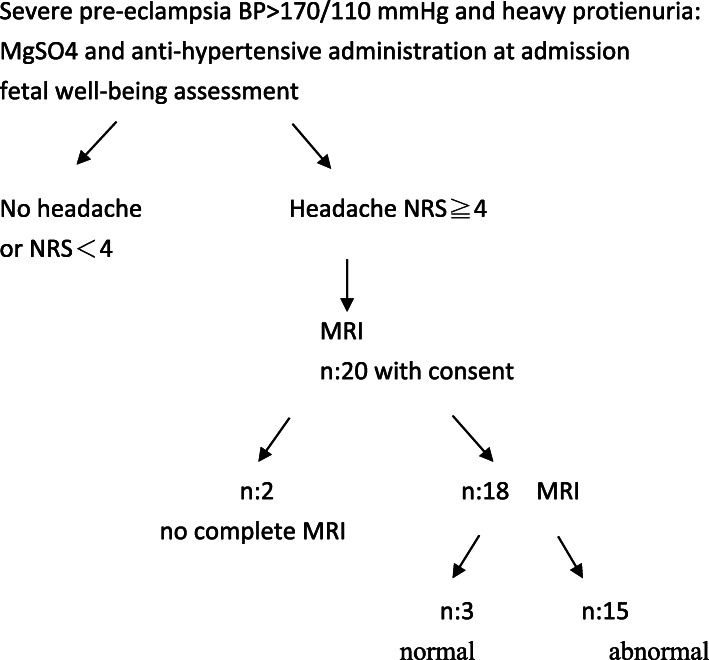
Algorithm for patient selection

In total, 20 consecutive patients were enrolled after written consent was obtained from them. The selection process is illustrated in Fig. 1. Two women were unable to complete the scanning because one had claustrophobia and one the other had several motion artifacts. Patient characteristics and clinical obstetrical parameters of all the 18 patients are presented in Table 1. All the women were Asian, and eight patients (50%) were nulliparous. One woman smoked tobacco and two had gestational diabetes mellitus. The mean age of the enrolled patients was 32 years (range, 25–40 years), with nine women aged > 34 years. The mean gestational age was 32 weeks (range, 29–38 weeks) and the mean body mass index was 30 (range, 22–44).

**Table 1:** Obstetrical Demographic Data

		N: 18
Mode of delivery		CS: 13
		SD: 5
Gravida		Nulliparous 8; Multiparous 8
Age(years, [Range])		32.2 (25–40)
BMI [Range]		30.3 (22–44)
Date of delivery (gestation week, [Range])		32 (29–38)
Preterm delivery		13/18^a^ one IUFD
Newborn Apgar score (min, [Range])		8 (7–10)
Newborn BW (min,[Range])		1850 (1005–2870)
IUGR (case number)	15/17:(83) %	15/17 (83%) with < 10% BW (8 /12); <5% BW (7/12) < 5% (7/12)

^a^ one IUFD (in-utero fetal demised), IUGR (intra-uterine growth restriction)

### Clinical presentation

All cases were enrolled via emergency referral, and fulfilled the criteria of severe pre-eclampsia with systolic blood pressure ≧ 160 mmHg or diastolic blood pressure ≧110 mmHg and headache, and associated with other CNS presentations such as visual disturbances or conscious deterioration. Systolic BP ranged from 160 to 180 mmHg in 16 women and was > 200 mmHg in four women at admission. The onset of clinical features occurred during the pre-delivery period, which contributed to high feto-maternal risks. Visual disturbances, presented as blur vision, occurred in these four patients. Seven patients had serum albumin level of < 2.5 mg/dL. Three patients had concurrent hemolysis, elevated liver enzymes, and low platelet count (HELLP syndrome). Neck stiffness and “tightness” discomfort were also noted in half of the patients. Headache intensity was recorded by subjective NRS and patients with a mean score of 5, were prescript pain relief medications. The headache was characteristically localized in the frontal and posterior occipital regions, as well in the upper neck. In this study, clinical features included headache (*n* = 18), blur vision (*n* = 2), vomiting (n = 2), and delirium (n = 1).

### MRI assessment

**Fig. 2:**
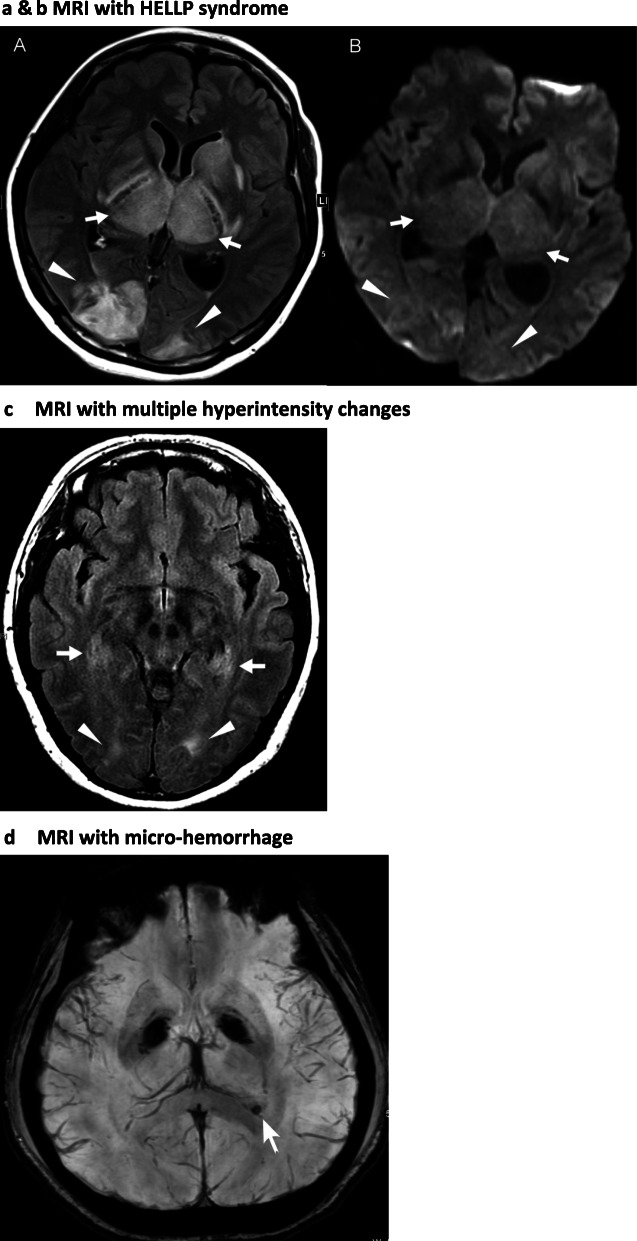
**a** & **b** MRI of patient with HELLP syndrome A 35-year-old pregnant women at 37 weeks of gestational experiencing severe pre-eclapmsia, headache and HELLP syndrome. Brain MRI revealing grossly symmetric hyperintensities over bilateral basal ganglia, thalami (white arrows) and occipital lobes (white arrowheads) on FLAIR, but minimal hyperintensities on DWI (b), consistent with typical PRES with vasogenic edema 2 (**c**) MRI with multiple hyperintensity changes Brain MRI of a 39-year-old pre-eclamptic pregnant women having headache at 28 weeks of gestation. Axial FLAIR disclosed symmetric hyperintensities on bilateral medial temporal lobes (white arrows) and occipital subcortical white matters (white arrowheads), suggestive of the mild form of PRES 2 (**d**) MRI with micro-hemorrhage A 25-year-old pregnant woman with gestational age of 26 weeks presented with headache and hypertension up to 200/100 mmHg. A profound hypointense focus on the periventricular white matter of left occipital horn was observed by SWI, indicating of a microhemorrhage (white arrow) and a precedent change of PRES onset

The typical imaging in a woman resulted in the identification of PRES with hyperintensities in the occipital, parietal, frontal, and temporal lobes and basal ganglia, brainstem, and cerebellum areas of the brain*.* In total, 12 patients had abnormal hyperintensities in the occipital lobes on FLAIR imaging. Among these 12 patients, 6 cases had localized abnormal subcortical hyperintensity confined in the occipital lobes, and 6 cases had abnormal subcortical hyperintensity in multiple temporal, parietal lobes, basal ganglia, and brainstem. Two patients had lesions on SWI, suggesting microhemorrhages in the occipital lobe. Only three patients had normal images. These MRI findings are listed in Table 2 and some notable MRI images of different patients are presented in Fig. 2a, b, c, and d.

**Table 2:** MRI hyperintensity changes in 18 cases

Localized area	n: 8
Occipital lobe	6 + (SWI in 2)^a^
Temporal lobe	0
Parietal lobe	0
Frontal lobe	0
Basal ganglia / cerebellum	0
**Multiple areas**	n: 7 (include one PRES)
Frontal lobe + Parietal lobe	1
Basal ganglia/cerebellum + Occipital lobe	0
Frontal lobe +Temporal lobe + Parietal lobe + Occipital lob	2
Temporal lobe + Occipital lobe	3
Cerebellum / brainstem + Basal ganglia / thalamus + Occipital lobe + Temporal lobe	1
**No abnormality**	n: 3

^a^*SWI* susceptibility-weighted imaging

### Obstetrical outcomes

Among the 18 patients, five had vaginal delivery. The cesarean section rate was 72%; nine patients had nonreassuring fetal heart rate, three had a cesarean section previously, and one had malpresentation. The mean gestational age at delivery was 32 weeks and 13 patients had preterm delivery, with one preterm delivery resulting in fetal death (Table 1). The mean interval from admission to delivery was 3 days (range, 1–9 days) and the mean length of postpartum stay was 4 days (range, 3–7 days). All the deliveries were live births with a 5-min Apgar score of > 7, except one wherein congenital anomaly resulted in an in utero death at 29 weeks of gestation. The newborns required no neonatal resuscitation and had no major postnatal complications. The mean birth weight was 1850 g (range, 1005–2870 g), with 15 cases (83%) small-for-date newborns (< 10 percentile) recorded.

### Postpartum recovery

After delivery, the average BP of women was controlled at 160/90 mmHg and the NRS of headache was < 3 within 24 to 48 h of delivery. They had no clinical deterioration to eclampsia or stroke and were discharged under normalcy. Normalcy was clinically defined as the absence of visual problems, headache, nausea/vomiting, or altered sensorium. Only one woman was still on anti-hypertensives 1 year after delivery.

## Discussion

### Principal findings of this study

The findings of this study suggested that symptomatic headache due to a hypertensive crisis in pregnancy is a reflection of early signs of cortical dysfunction that can be noted as abnormal cortical hyperintensities on MRI scanning. Posterior reversible encephalopathy syndrome (PRES) is also known as acute hypertensive encephalopathy or reversible posterior leukoencephalopathy. Therefore the imaging presentation of cortical change on MRI is focused on acute changes and is different from chronic hypertensive encephalopathy. Cortical changes resulted in primary CNS injuries are common in women (more than 80%) experiencing severe preeclampsia with headache. The findings of this study not only revealed that early MRI changes such as abnormal T2-wieighted imaging and FLAIR intensities at the relevant sites are correlated with PRES; this supports the hypothesis that PRES can be present as an antecedent to eclamptic seizure.

### Results in the context of other studies

PRES is a radiological diagnosis, not isolated to one clinical entity or diagnosis, and is most commonly reported in patients with hypertensive encephalopathy [10–12]. Most retrospective studies on PRES complicating pregnancy and the puerperium period have focused on exploring the relationship between eclampsia and the concurrence of PRES [8, 13]. Differentiating the underlying etiology of PRES based on imaging alone is not possible; however, gender-specific conditions in reproductive age women experiencing preeclampsia, severe gestational hypertension, and eclampsia are the most commonly encountered, such women are particularly susceptible to PRES [14, 15]. The radiation exposure of catheter angiography is the major concern for pregnant women and is avoid in our clinical practice. As severe hypertension with autoregulatory failure and forced hyper-perfusion remains the most widely accepted mechanism of PRES (8). The endothelial dysfunction seen in preeclampsia might be demonstrated using a new non-invasive MRI technique, Intravoxel incoherent motion (IVIM), for assessment of cerebral capillary blood perfusion. Hypoperfusion was detected at only a portion of the basal ganglia in preeclampsia but not in normal pregnancy or non-pregnant women [16]. The autoregulatory response is intended to maintain stable cerebral blood flow in the face of blood pressure fluctuations. With acute severe hypertension, precapillary arteriolar vasoconstriction occurs in small (30–300 μ) resistance vessels that limit cerebral blood flow. The diagnostic performance of MR arteriogram (MRA) for such small-size arteries is very limited and not mentioned for diagnosis of PRES [5]. The pregnant women in this study had acute hypertension, usually at uncomfortably status, MRI scans protocol were designed as short acquisition time as possible to avoid motion artifacts and no need sedation medication, MRA being omitted thereof.

In last decade, eclampsia was identified as the inciting factor in 20% cases of PRES [17]; however in a recent report, women with eclampsia had an extremely high risk (more than 90%) of developing PRES among all patients [3]. Only one study on PRES in a few patients with severe preeclampsia had demonstrated that it precedes eclamptic convulsion [3]. The present prospective study aimed to determine whether and how the clinical and CNS imaging findings of patients having severe preeclampsia with onset of a significant headache were related to PRES using MRI.

Women with PRES during pregnancy predominantly have neurological symptoms such as white matter cerebral edema on computed tomography and MRI scans because of a loss in autoregulatory capacity, blood–brain barrier (BBB) disruption, and subsequent vasogenic edema surrounding cerebral arteries and arterioles [18, 19]. Diffusion-weighted imaging (DWI) had a greater ability to distinguish between vasogenic and cytotoxic edema in PRES [18, 20]. Typical neuroimaging studies usually showed bilateral and symmetric brain edema in subcortical regions of the parietal and occipital lobes (98%), wherein vasogenic edema rather than cytotoxic edema may be pivotal in neuroimaging studies [3, 8, 21]. However, the lesions can also extend to other brain structures such as the frontal lobes (68%), temporal lobes (40%), cerebellar hemispheres (30%), basal ganglia (14%), brain stem (13%), and deep white matter, particularly the splenium of the corpus callosum (10%). Unilateral involvement was relatively infrequent (28%) in this study. Only one patient had headache with altered conscious status; MRI revealed typical bilateral PRES (Fig. 2a and b). In addition, two patients presented SWI in the occipital lobe which suggested micro-hemorrhages (Fig. 2d). To differential diagnosis between cerebral venous sinus thrombosis and other venographic abnormality, MRI scans usually presenting unilateral local edema, petechial or frank confluent hemorrhage at the affected region, not cortical microbleeds [22]. The clinical presentations were crucial when micro-hemorhages were suspected; BP and neurological examinations of two patients were closely monitored, and were found to be stable from the admission to delivery.

Of the 15 patients, 12 patients had abnormal images that appeared as iso-intense or had low signal intensity on T1-weighted images (T1WI), and high signal intensity on T2-weighted images (T2WI) and FLAIR (Table 2 and Fig. 2c). T2WI and FLAIR images showed hyperintensities within cerebral white matter (white matter hyperintensities [WMH]) or subcortical gray matter (gray matter hyperintensities [GMH]) in young women, which can be for various reasons, including ischemia, microhemorrhages, gliosis, damage to small blood vessel walls, barrier breaches between the cerebrospinal fluid and the brain, or loss and deformation of the myelin sheath [12, 13, 19]. However, during pregnancy and complicated with severe preeclampsia, hyperintensity on FLAIR is the most common and typical finding of PRES. The hyperintensity sites in this study were comparable to those in PRES, with the occipital lobes most commonly involved [21]. Focal areas included symmetric multilobar/hemispheric edema with predominant involvement of the parietal and occipital lobes, with the cerebellum being less commonly involved. These pictures were also revealed in our study (Table 2), further demonstrating that MRI could reveal early crucial CNS changes of PRES in women with severe preeclampsia.

SWI played a significant role in detecting microbleeds in women with PRES, which can be easily missed in conventional T1WI, T2WI, and FLAIR. McKinney et al. reported the role of SWI in PRES; however, studies on severe preeclamptic status–related PRES were not available [23, 24]. In addition, WMH on FLAIR at bilateral trigones should be carefully scrutinized, which can be easily overlooked as normal presentation if no history of acute hypertension in early PRES presentation has been provided. Under such circumstances [25], this current study provide evidence for progression of PRES to ischemia or hemorrhage [23, 25] through neuroimaging.

All the 18 cases had hypoalbuminemia and some had serum albumin levels of ≤2.5 mg/dl as a result of protein loss because of heavy proteinuria. Such fluid retention and imbalances can predispose a patient to PRES via blood pressure lability and elevation, which explains the exacerbated subcortical hyperintensity on CNS imaging in patients with hypoalbuminemia and HELLP revealed [1, 19]. Extensive involvement was noted among three patients with HELLP in our study based on prominent subcortical hyperintensities sites (Fig. 2a and b). Our cases supported this constitution that endothelial dysfunction increased vulnerability of the cerebral vasculature to loss of autoregulation and increased permeability of the blood–brain barrier predisposed by severe pre-eclampsia [12, 19].

The clinical presentation in PRES may be nonspecific, but variable alterations in consciousness, headaches, and visual disturbances, as well as radiological findings of focal reversible vasogenic edema [11, 24, 26]. The onset of symptoms is usually subacute without prodrome that develops within 12–48 h or even several days, but patients with severe pre-eclampsia must be cautious because the clinical course can be rapid deterioration into eclampsia. Special precaution for BP must be taken when headache causes sleeping or daily activities disturbance in pregnant women. Our patients did not exhibit restlessness, agitation or convulsion throughout the hospitalization period. The mild neck stiffness, or tightness, without association of CNS infection, was resolved after blood pressure was controlled. Involvement of either the optical tracts/nuclei or the parieto-occipital cortices was not demonstrated by the MRI, and the visual symptoms resolved within 2–3 days after delivery. Our ophthalmologists examined eye grounds of each case during hospitalization and no macular lesions or retinal detachment was reported. However, no routine follow-up of ophthalmology assessment was arranged due to prompt recovery.

### Strengths and limitations

This study revealed two significant findings. First, advanced neuroimaging techniques such as MRI indicated the changes in subcortical regions were comparable to those of PRES in most of patients (≥80%) having severe pre-eclampsia combined with headache. Second, SWI could identify microhemorrhage in patients, and prompt management could prevent deterioration into eclampsia or stroke. Our findings had assumptions related to the precedence of vasogenic edema in PRES, which was a delayed imaging picture of severe cortical dysfunction, such as eclampsia. This cohort study focused on the influence of acute hypertension, a transient and reversible phenomenon, on pre-eclampsia. The sample size and non-randomized methods were the main limitations of this study. No electroencephalography or follow-up MRI were performed to evaluate cerebral functions and long-term consequences of severe preeclamptic women with abnormal imaging [3, 6, 10], however their headache subsided within 2 days after delivery.

## Conclusion

This study aimed to detect and investigate, using MRI, the pathophysiology of early CNS-related in women with severe pre-eclampsia with headache. Our study results suggest that PRES was an infrequent association without eclampsia. Moreover, there are changes of cortical hyperintensity at the regions comparable to those of PRES Persisting headache in women with severe pre-eclampsia provides an indication for performing MRI scan to exclude the presence of CNS-related pathological conditions. The present management of severe pre-eclampsia remains the golden standard for the prompt control of hypertensive crisis. Furthermore, adding a MRI to diagnostic procedures can facilitate determining the condition severity, thus allowing appropriate work-up to forestall serious potential sequelae, because proper management with a timely delivery can prevent eclampsia and result in favorable maternal and fetal outcome.

## Data Availability

The datasets used and analyzed in this study are available from the corresponding author on reasonable request.

## Abbreviations

MRI: Magnetic resonance imaging
PRES: Posterior reversible encephalopathy syndrome
BBB: Blood brain barrier
BP: Blood pressure
FLAIR: Fluid attenuated inversion recovery
DWI: Diffusion-weighted imaging
SWI: Susceptibility-weighted imaging
